# Documentation of Behavioral Health Risk Factors in a Large Academic Primary Care Clinic

**DOI:** 10.1177/21501319221074466

**Published:** 2022-03-30

**Authors:** Stephanie Hosang, Natasha Kithulegoda, Noah Ivers

**Affiliations:** 1 University of Toronto, Toronto, ON, Canada; 2 Women’s College Hospital, Toronto, ON, Canada

**Keywords:** primary care, chart review

## Abstract

**Objective:**

To determine the prevalence of alcohol, smoking, and physical activity status documentation at a family health team in Toronto, Ontario, and to explore the patient characteristics that predict documentation of these lifestyle risk factor statuses.

**Design:**

Manual retrospective review of electronic medical records (EMRs).

**Setting:**

Large, urban, academic family health team in Toronto, Ontario.

**Participants:**

Patients over the age of 18 that had attended a routine clinical appointment in March, 2018.

**Main Outcome Measures:**

Prevalence and content of risk factor status in electronic medical records for alcohol, smoking, and physical activity.

**Results:**

The prevalence of alcohol, smoking, and physical activity documentation was 86.4%, 90.4%, and 66.1%, respectively. These lifestyle risk factor statuses were most often documented in the “risk factors” section of the EMR (83.7% for alcohol, 88.1% for smoking, and 47.9% for physical activity). Completion of a periodic health review within 1 year was most strongly associated with documentation (alcohol odds ratio [OR] 9.79, 95% Confidence Interval [CI] 2.12, 45.15; smoking OR 1.77 95% CI 0.51, 6.20; physical activity OR 3.52 95% CI 1.67, 7.40).

**Conclusion:**

Documentation of lifestyle risk factor statuses is strongly associated with having a recent periodic health review. If “annual physicals” continue to decline, primary care providers should final additional opportunities to address these key determinants of health.

## Introduction

Population surveys indicate that Canadians continue to exhibit unhealthy lifestyle behaviors. 15% of Canadians drink above Canada’s Low-risk Alcohol Drinking Guidelines, 15% of Canadians use tobacco products on a regular basis, and only 16% of Canadian adults meet national physical activity guidelines of 150 min of moderate-vigorous activity per week.^1-4^ This represents over 50 million Canadians who continue to engage in unhealthy behaviors. Additionally, there is tremendous mortality, morbidity, and economic cost associated with these behaviors. Smoking continues to be the number one cause of preventable disease and death in Canada, with more than 72% of lung cancer cases being secondary to smoking.^5^ Each year there are more hospitalizations from alcohol than from heart attacks, and this has increased in the context of the current COVID-19 pandemic.^6^

Primary care providers (PCPs) are uniquely positioned to impact the health behaviors of their patients.^7^ Unfortunately, previous research has shown poor documentation of risk factors such as alcohol and tobacco.^8^ It is difficult to track and monitor treatments for conditions if they are not documented accurately in the medical record. As such, improving the quality of medical documentation may lead to improvements in quality of care.^9^ The increasing use of electronic medical records (EMRs) among Canadian primary care providers may serve as a catalyst for such improvements.^10,11^ For example, it could enable use of computerized decision support systems, which are known to improve guideline concordant processes of care.^12–14^

While improving documentation of risk factors in EMRs represents a useful step on a quality improvement journey, there is limited information about where to begin: little is known about the patient characteristics associated with documentation of risk factors in primary care EMRs. Understanding the predictors of better and worse documentation may help inform future interventions aiming to improve documentation and address these risk factors in primary care. Therefore, we sought to (i) determine the extent and nature of current risk factor documentation and (ii) assess patient characteristics that predict documentation of alcohol, smoking, and physical activity status in the electronic medical record.

## Methods

### Study Design and Participants

A retrospective EMR review was performed in the Women’s College Hospital Family Practice Health Centre (WCH FPHC), an academic family practice affiliated with Women’s College Hospital. Patients that attend this clinic are patients of primary care providers, accessing primary care services. WCH FPHC was chosen as results of this study will be used to inform a larger, quality improvement study on documentation in WCH FPHC. EMR following a protocol reviewed and approved by the WCH Research Ethics Board.

EMRs were reviewed for patients over the age of 18 that had attended a clinical appointment at 9:00 am or 1:00 pm with a staff physician at Women’s College Hospital Family Practice (WCHFP) between March 1st, 2018 and March 31st, 2018. These appointment times were chosen arbitrarily for logistical reasons; we had no reason a priori to believe that they would be used by patients that were systematically different. Appointments involving a periodic health review (ie, “annual physical”), or urgent care were not reviewed. We chose to focus on routine office visits because we believe these are the visits where future interventions would be most easily implemented. There were no other exclusion criteria.

### Data Collection and Management

EMRs were manually reviewed on site by members of the research team. A manual EMR review was necessary because physicians document lifestyle risk factor status and treatment information in various sections of the patient EMR. Extracted data (if available) included demographic information (age, gender, body mass index [BMI], socioeconomic status based on income quintile), lifestyle risk factors (alcohol, smoking, physical activity), follow-up treatment information (prescription or referral for alcohol use disorder, smoking cessation, and/or physical activity), EMR location of lifestyle risk factor statuses and follow-up treatment information, and indicators of overall health (periodic health review in the last year, number of current prescribed medications, number of hospitalizations in the last year, number of emergency room visits in the last year).

### Outcomes

Primary outcomes were documentation of risk factor status for alcohol, smoking, and physical activity. Secondary outcomes were the presence and content of follow-up treatment information (prescriptions for alcohol use disorder, smoking cessation, and/or physical activity; referrals for alcohol use disorder, smoking cessation, and/or physical activity), and the location of the outcomes in the EMR. Clinical severity of the risk factor status was also assessed. Alcohol status was risk stratified based on the Alcohol Use Disorders Identification Test (AUDIT).^15^ Physical activity status was stratified based on achievement of the Canadian Physical Activity Guidelines recommendation of at least 150 min of moderate-vigorous intensity physical activity per week.^7^

### Statistical Analysis

Data were analyzed using SPSS. For each of the primary outcomes, a hierarchal logistic regression was performed to assess the association between patient-level factors and documentation status, accounting for the primary care provider of each patient. The covariates included: age, sex, body mass index, estimated neighborhood income quintile calculated via postal code,^16^ periodic health review within the last year, number of current prescribed medications, number of hospitalizations in the last year, number of emergency room visits in the last year, and primary care provider ID. Since 57 patients were missing body mass index data (height and weight) in their EMR, we decided to perform 2 sets of logistic regression analyses: primary analyses without body mass index as a covariate, and sensitivity analyses that replaced missing body mass index data with the median value.

## Results

### Patient Characteristics

Table 1 shows characteristics of the 177 included patients. Ages ranged from 19 to 92 years, with a mean age of 52 (SD = 16.6). The majority of patients were female (74.6%; n = 132). The majority of patients (31.1%; n = 55) were within the highest income quintile. Seventy-eight patients (44.1%) had completed a periodic health review in the prior year. Most patients were relatively healthy: 155 (87.6%) had no hospitalizations in the prior year, 146 (82.5%) had no emergency room visits in the prior year, and 92 (52.0%) were taking less than 5 prescribed medications.

**Table 1.** Characteristics of Patients in EMR Review (n = 177).

	Mean	SD	Range
Age	52.04	16.62	19-92
Sex	n	%	
Female	132	74.6	
Male	45	25.4	
Body mass index			
<18.5	4	2.3	
18.4-24.9	23	13.0	
25.0-29.9	36	20.3	
30.0-34.9	24	13.6	
35.0-39.9	5	2.8	
Not documented	85	48.0	
Estimated neighborhood income quintile			
1 (lowest)	25	14.1	
2	30	16.9	
3	29	16.4	
4	31	17.5	
5 (highest)	55	31.1	
Not documented	7	4.0	
Periodic health review			
Completed within last year	78	44.1	
Not completed within last year	99	55.9	
Number of current prescribed medications			
0-4	92	52.0	
5-10	68	38.4	
More than 10	17	9.6	
Number of hospitalizations within the last year			
0	155	87.6	
1-3	22	12.4	
Number of ER visits within the last year			
0	146	82.5	
1-6	31	17.5	

### Lifestyle Status Documentation

Table 2 shows the prevalence of lifestyle risk factor status documentation for alcohol, smoking, and physical activity.

**Table 2.** Lifestyle Status Documentation (n = 177).

	n	%
Alcohol documentation		
Documented	153	86.4
Low risk	117	76.0
Medium risk	23	14.9
High risk	11	7.1
Likely alcohol use disorder	3	1.9
Not documented	24	13.6
Location in EMR		
Risk factors	128	83.7
Appointment notes	24	15.7
Dietician notes	1	0.7
Prescriptions to reduce alcohol consumption	3	2.0
Smoking documentation		
Documented	160	90.4
Never smoked	117	73.1
Current smokers	9	5.6
Ex-smokers	34	21.3
Not documented	17	9.6
Location in EMR		
Risk factors	141	88.1
Appointment notes	19	11.9
Prescriptions for smoking cessation	2	1.3
Physical activity documentation		
Documented	117	66.1
Meeting guidelines	41	35.0
Not meeting guidelines	76	65.0
Not documented	60	33.9
Location in EMR		
Risk factors	56	47.9
Appointment notes	54	46.2
Dietician notes	3	2.6
Physician reminders	4	3.4
Prescriptions to increase physical activity	4	3.4

Most patient EMRs (86.4%; n = 153) had a documented alcohol status and most commonly (87.3%; n = 128) this was identified in the risk factors section of the patient EMR. Of those with alcohol status documented, 76.0% (n = 117) of patients were stratified as low risk, 14.9% (n = 23) as medium risk, 7.1% (n = 11) as high risk, and 1.9% (n = 3) as likely alcohol use disorder. Each of the patients with likely alcohol use disorder had prescriptions to address alcohol use. No patients had documented referrals to a specialist for alcohol use.

Nearly all (90.4%; n = 160) patient EMRs had a documented smoking status, most commonly (88.1%; n = 141) recorded in the risk factors section of the EMR. Of those with smoking status documented, 73.1% (n = 117) had never smoked, 5.6% (n = 9) were current smokers, and 21.3% (n = 34) were ex-smokers. Only 2 of 9 current smokers had documented prescriptions. No patients had documented referrals to a specialist for smoking cessation.

Many patient EMRs (66.1%; n = 117) had a documented physical activity status. This was occasionally (47.9%; n = 56) documented in the risk factors section of the EMR, but just as commonly (46.2%; n = 54) recorded in the progress notes. Of those with physical activity documentation, 35.0% of patients (n = 41) met the recommended Canadian Physical Activity Guidelines of at least 150 min of moderate-vigorous intensity physical activity per week.^7^ Only 3.4% (n = 4) of patients had documented prescriptions to increase physical activity. No patients had documented referrals to a specialist for physical activity.

### Predictors of Lifestyle Status Documentation

Table 3 shows the results of the primary logistic regression analyses performed. The logistic regression model for alcohol documentation explained 19.1% (Nagelkerke *R*^2^) of the variance in alcohol documentation. Those with a periodic health review in the prior year had 9.79 greater odds for documentation of alcohol status than those without. Other covariates did not add significantly to the model. The regression model for smoking documentation only explained 4.5% of the variance in smoking documentation. No covariates significantly contributed to the model. The regression model for physical activity documentation explained 16.4% of the variance in physical activity documentation. Those with a periodic health review in the prior year had 3.51 greater odds to have physical activity documentation than those without. Other covariates did not add significantly to the model. The sensitivity analysis did not lead to different results.

**Table 3.** Predictors of Lifestyle Status Documentation (n = 170).

	Odds ratio	95% confidence interval	*P* value
Alcohol documentation			
Age	0.98	0.95, 1.01	.21
Sex	0.46	0.14, 1.56	.21
Income quintile	0.89	0.63, 1.27	.51
Periodic health review	9.79	2.12, 45.15	.00
Medications	1.04	0.90, 1.20	.58
Hospitalizations	1.44	0.51, 4.07	.49
ER visits	0.70	0.40, 1.22	.20
Smoking documentation			
Age	1.00	0.96, 1.04	.99
Sex	1.84	0.55, 6.15	.32
Income quintile	1.17	0.79, 1.75	.43
Periodic health review	1.77	0.51, 6.20	.37
Medications	1.04	0.87, 1.23	.68
Hospitalizations	0.78	0.30, 2.07	.62
ER visits	0.82	0.40, 1.69	.60
Physical activity documentation			
Age	1.01	0.99, 1.03	.37
Sex	0.52	0.23, 1.20	.13
Income quintile	0.83	0.65, 1.05	.12
Periodic health review	3.52	1.67, 7.40	.00
Medications	0.97	0.87, 1.07	.52
Hospitalizations	0.63	0.34, 1.18	.15
ER visits	1.02	0.62, 1.67	.94

## Discussion

Our findings suggest that lifestyle risk factor status for alcohol, smoking, and physical activity are well-documented in EMRs of a healthy, convenience-sampled cohort of patients in an academic family practice, and that documentation of lifestyle risk factor statuses is strongly associated with having a recent periodic health review. Alcohol and smoking statuses were reported in 86.4% and 90.4% of patient EMRs, respectively, a rate higher than that of previous Canadian studies.^9,10^ It has been suggested that documentation rates are higher among provider’s primary roster and in academic centers.^16,17^ The interplay of both of these factors may have contributed to higher rates of documentation among our cohort. Additionally, previous studies investigating documentation of cardiovascular risk factors including blood pressure, physical activity, and smoking status have reported physical activity as the least often documented lifestyle factor,^18,19^ a finding reproduced in our results. This highlights the need for improvement in documentation of protective lifestyle factors, and not just those that increase risk of chronic disease.

Our findings that 23.9% of patients would be classified as medium-high risk (ie, drinking greater than Canada’s Low Risk Drinking Guidelines^1^) is consistent with national estimates from 2014 which reported that 18% of Canadians engaged in risky drinking behaviors.^20^ However, we found a lower prevalence of smoking (5.6% current smokers, 21.3% ex-smokers) when compared to national estimates from 2017, which identified 15.1% of Canadians as current smokers and 63.1% as ex-smokers.^3^ With respect to physical activity, 35.0% of patients were meeting physical activity guidelines, which is greater than the 16% of Canadian adults meeting guidelines in 2020.^4^ The overall similarities between our data and national estimates support that lifestyle statuses were well-documented in our setting, and that our population’s lifestyle behaviors were similar to that of the general population.

Location of lifestyle risk factor documentation was varied, with the risk factors section being the most common location to record statuses. Interestingly, location of physical activity statuses was the most varied among the 3 risk factors with statuses located in the risk factors section, appointment notes, dietician notes, and provider reminders sections of patient EMRs. A study investigating the quality of medical documentation among 834 patients in Massachusetts, found that quality of medical documentation was largely dependent upon the measure in question.^10^ The variability in location of statuses among our primary outcomes supports this, and points to the need for interventions that standardize documentation across risk factors.

The main predictor of lifestyle risk factor documentation was completion of a recent periodic health review. Lifestyle habits are an important component of many Preventative Care Checklist Forms that guide periodic health review visits.^19^ The association between recent periodic health review and documentation may indicate that it is easier to address lifestyle factors in a periodic health review in comparison to routine visits where other concerns may take precedent. As family medicine moves toward fewer “annual physicals,”^21^ it will become increasingly important to find new ways of incorporating lifestyle screening and documentation into routine visits.

Standardization of EMR documentation across practices with diagnostic tools such as the AUDIT, may help to more easily track lifestyle risk factors, and in turn lead to improvements in care. The need for improved documentation accuracy is highlighted in 1 study on alcohol documentation that reported that improved documentation did not necessarily reflect patient reports of alcohol counseling or decreased alcohol use.^16^ The development of digital screening tools that provide counseling reminders to PCPs may be a way to address this disconnect. Such interventions may also help to incorporate screening into visits other than the “annual physical,” which may become decreasingly popular in the coming years.^22^

We acknowledge some important limitations of this study. Mainly, the small sample size of patient EMR reviews from a convenience sample within a single urban academic family practice make the prevalence of lifestyle risk factor documentation difficult to generalize. In particular, data examined a cohort of mainly healthy, mostly female patients living in high-income neighborhoods. Still, it is reassuring that the prevalence of alcohol, smoking, and physical activity behaviors are in line with that of the general population. Further, our models predicted only a small proportion of the variation in documentation. Future studies should aim to identify other patient- and provider-related characteristics that predict documentation in larger and randomly selected patient populations.

## Conclusions

This study adds to the literature on the documentation of lifestyle risk factors among primary care providers. It demonstrates that although documentation may be improving at some primary care sites, there is a need for improved documentation accuracy and standardization among providers. Furthermore, the results indicate that documentation can be predicted based on completion of a recent periodic health review. This highlights the need for primary care providers to target these behaviors at other times. Additionally, this study supports the need for interventions that standardize documentation, encourage counseling in routine visits, and identify patients in need of further treatment.

## References

[1] Stockwell T Beirness D Butt P Gliksman L Paradis C . Canada’s low-risk drinking guidelines. CMAJ. 2012;184(1):75. 10.1503/cmaj.112-2007 PMC3255216 22232339

[2] Canadian Centre on Substance Use and Addiction (CCSA). Canadian Drug Summary: Alcohol. CCSA; 2019.

[3] Reid JL Hammond D Tariq U Burkhalter R Rynard VL Douglas O . Tobacco Use in Canada: Patterns and Trends, 2019 Edition. Propel Centre for Population Health Impact, University of Waterloo; 2019.

[4] Centre for Surveillance and Applied Research, Public Health Agency of Canada. Physical Activity, Sedentary Behaviour and Sleep (PASS) Indicators Data Tool, 2020 Edition. Public Health Infobase, PHAC; 2020.

[5] The BREATHE Lung Association. Smoking and Tobacco Statistics. Canadian Lung Association; 2021.

[6] Government of Canada. Canadian Tobacco and Nicotine Survey: Summary of Results for 2019. Statistics Canada; 2019.

[7] Canadian Society for Exercise Physiology. Canadian physical activity guidelines. 2020. Accessed August, 2020. https://csepguidelines.ca/

[8] Bardach SH Schoenberg NE . The role of primary care providers in encouraging older patients to change their lifestyle behaviors. Clin Gerontol. 2018;41(4):326-334. 29221431 10.1080/07317115.2017.1376029 PMC5893434

[9] Dubey V Mathew R Iglar K Moineddin R Glazier R . Improving preventive service delivery at adult complete health check-ups: the preventive health evidence-based recommendation form (PERFORM) cluster randomized controlled trial. BMC Fam Pract. 2006;7:44. 16836761 10.1186/1471-2296-7-44 PMC1543627

[10] Soto CM Kleinman KP Simon SR . Quality and correlates of medical record documentation in the ambulatory care setting. BMC Health Serv Res. 2002;2:22-29. 12473161 10.1186/1472-6963-2-22 PMC140026

[11] Biro SC Barber DT Kotcha JA . Trends in the use of electronic medical records. Can Fam Physician. 2012;58:e21. PMC3264034 22267635

[12] Garg AX Adhikari NKJ McDonald H , et al. Effects of computerized clinical decision support systems on practitioner performance and patient outcomes: a systematic review. JAMA. 2005;293(10):1223-1238. 15755945 10.1001/jama.293.10.1223

[13] Souza NM Sebaldt RJ Mackay JA , et al. Computerized clinical decision support systems for primary preventive care: a decision-maker-researcher partnership systematic review of effects on process of care and patient outcomes. Implement Sci. 2011;6:87. 21824381 10.1186/1748-5908-6-87 PMC3173370

[14] Babor TF Higgins-Biddle JC Saunders JB Monteiro MG . AUDIT: The Alcohol Use Disorders Identification Test. World Health Organization; 2001.

[15] *Postal CodeOM Conversion File (PCCF)* , 2017. Statistics Canada Catalogue no. 92-154-X.

[16] Berger D Lapham GT Shortreed SM , et al. Increased rates of documented alcohol counseling in primary care: more counseling or just more documentation? J Gen Intern Med. 2017;33(3):268-274. 29047076 10.1007/s11606-017-4163-2 PMC5834950

[17] McBride P Plane M Underbakke G Brown R Solberg L . Smoking screening and management in primary care practices. Arch Fam Med. 1997;6(2):165-172. 9075453 10.1001/archfami.6.2.165

[18] Tahaineh L Barakat S Albsoul-Younes AM Khalifeh O . Primary prevention of cardiovascular disease in a primary care setting. Prim Health Care Res Dev. 2016;17:311-316. 26621005 10.1017/S1463423615000559

[19] Ludt S Petek D Laux G , et al. Recording of risk-factors and lifestyle counselling in patients at high risk for cardiovascular diseases in European primary care. Eur J Prev Cardiol. 2011;19(2):258-266. 21450582 10.1177/1741826711400510

[20] Public Health Agency of Canada. Alcohol Consumption in Canada: The Chief Public Health Officer’s Report on the State of Public Health in Canada 2015. PHAC; 2016.

[21] Ridley J Ischayek A Dubey V Iglar K . Adult health checkup: update on the preventive care checklist form. Can Fam Physician. 2016;62:307-313. 27076540 PMC4830652

[22] Ponka D . The periodic health examination in adults. CMAJ. 2014;186(16):1245. 25288315 10.1503/cmaj.141125 PMC4216260

